# Supplementary material for: Charge redistribution of a spatially differentiated ferroelectric Bi4Ti3O12 single crystal for photocatalytic overall water splitting
Source: Nat Commun. 2024 Jun 4;15:4746. doi: 10.1038/s41467-024-49168-2 (PMC11150255; doi:10.1038/s41467-024-49168-2)
Supplement: Supplementary file 1 — Supplementary Information [file 41467_2024_49168_MOESM1_ESM.pdf]

Supplementary Information

Charge re-distribution of a spatially differentiated ferroelectric $Bi_4Ti_3O_{12}$ single crystal for photocatalytic overall water splitting

Guangri Jia[1, 2, 6], Fusai Sun[3, 6], Tao Zhou[2], Ying Wang[4], Xiaoqiang Cui[5], Zhengxiao Guo[2]*, Fengtao Fan[3]*, and Jimmy C. Yu[1]*

[1] Department of Chemistry, The Chinese University of Hong Kong, Shatin, New Territories, Hong Kong 999077, China

[2] Department of Chemistry, The University of Hong Kong, Pokfulam Road, Hong Kong 999077, China

[3] State Key Laboratory of Catalysis, Dalian National Laboratory for Clean Energy, iChEM, Dalian Institute of Chemical Physics, Chinese Academy of Sciences, Dalian 116023, China

[4] Department of Applied Biology and Chemical Technology, The Hong Kong Polytechnic University, Hung Hom, Kowloon, Hong Kong SAR, China

[5] State Key Laboratory of Automotive Simulation and Control, School of Materials Science and Engineering, Key Laboratory of Automobile Materials of MOE, Jilin University, Changchun 130012, China

[6] These authors contributed equally to this work.

[*] Corresponding emails: jimyu@cuhk.edu.hk (J. C. Yu), zxguo@hku.hk (Z. Guo) and ftfan@dicp.ac.cn (F. Fan)

Supplementary Figures

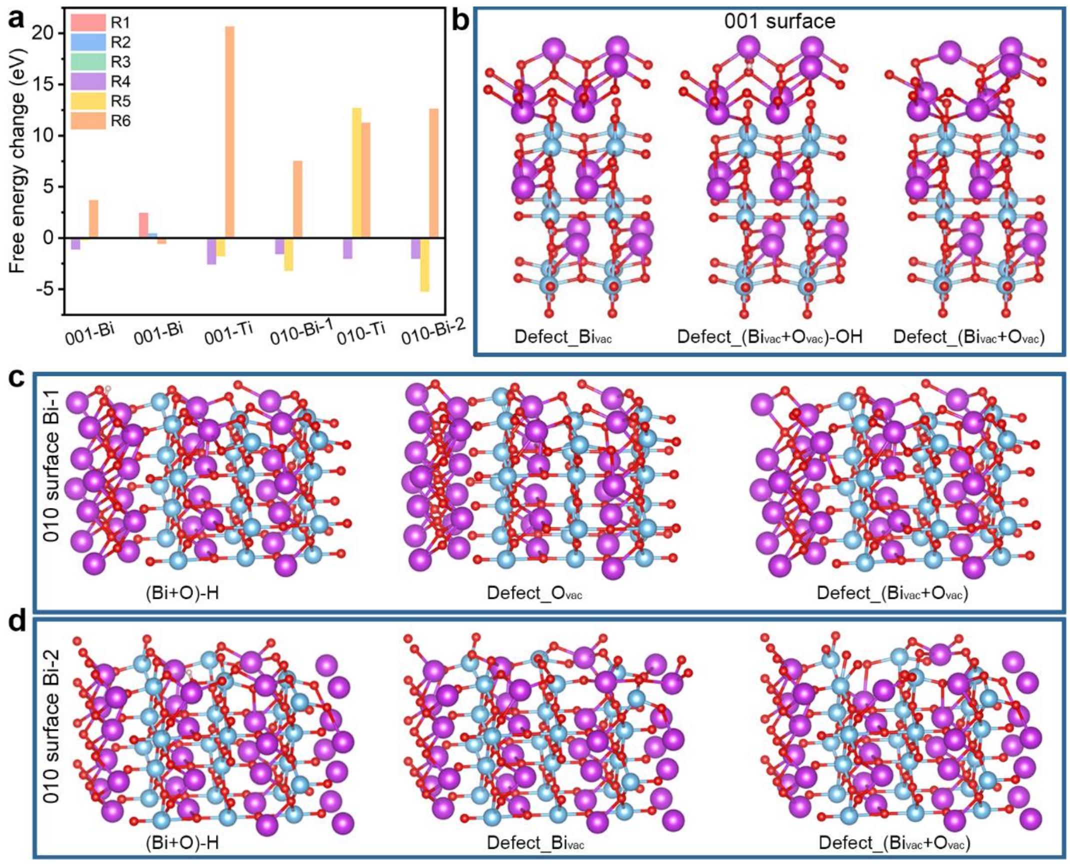

Supplementary Fig. 1. a) Free energy change of each step in the acid-etching reaction. **b**–**d**) Structure evolution in the acid-etching reaction. R1–R3 is the M-pathway (metal dissolution). R4–R6 is the O-pathway (oxygen dissolution). According to the free energy change, the stability: $Bi_{(001)}$ (ΔG_{max} = 3.17 eV) < $Bi_{(010)}$ (ΔG_{max} = 12.68 eV) < $Ti_{(001)}$ (ΔG_{max} = 19.62 eV). The 010-Bi-1 and 010-Bi-2 represent the different Bi site bonded with O on (010) surface, respectively.

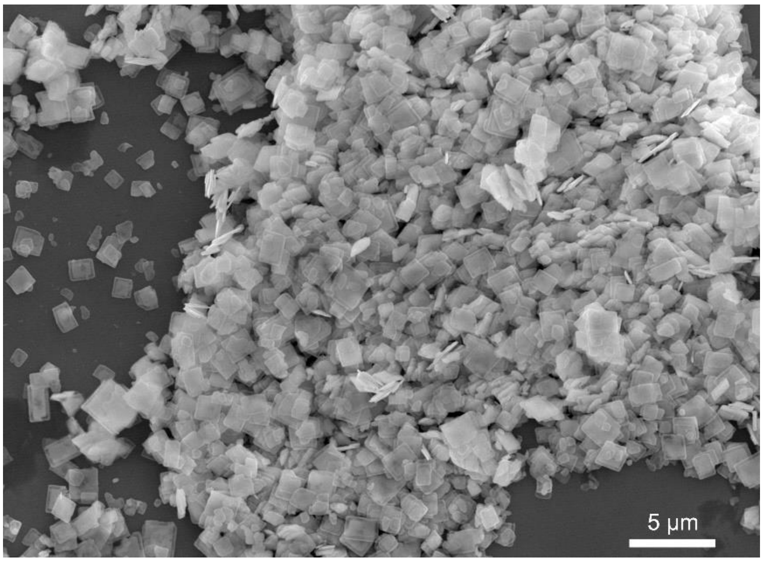

Supplementary Fig. 2. The large-scale SEM image of HCl treated duration for 2 h.

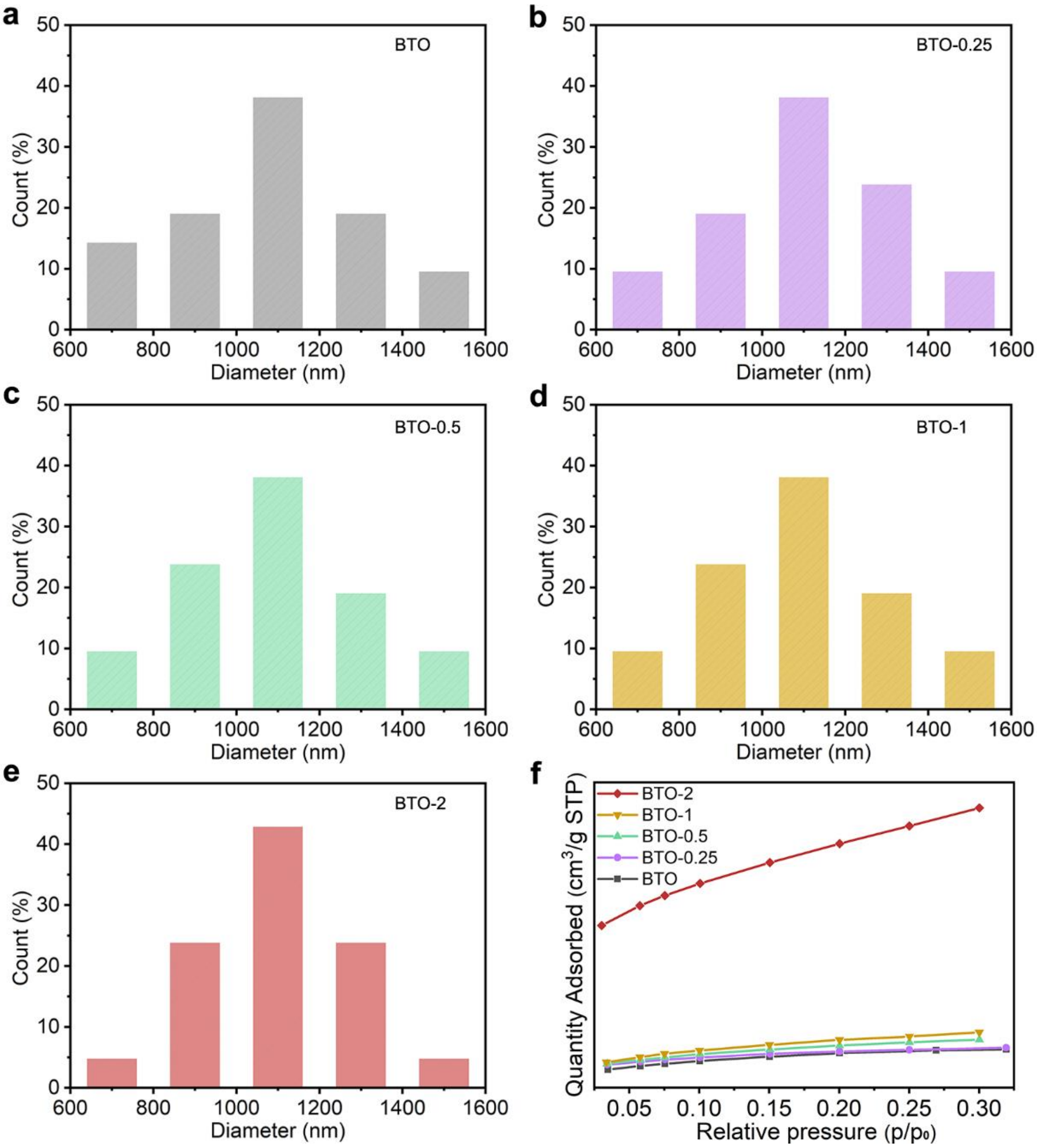

Supplementary Fig. 3. The particle size of basal surface of samples with and without acid etching: **a**) BTO, **b**) 0.25 h, **c**) 0.5 h, **d**) 1 hours, and **e**) 2 h. **f**) The N_2 adsorption isotherms plots.

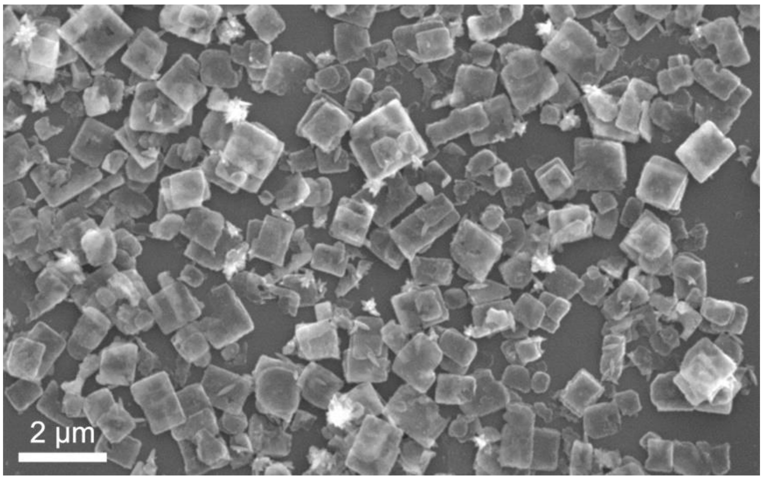

Supplementary Fig. 4. The SEM image of HCl treated duration for 4 h.

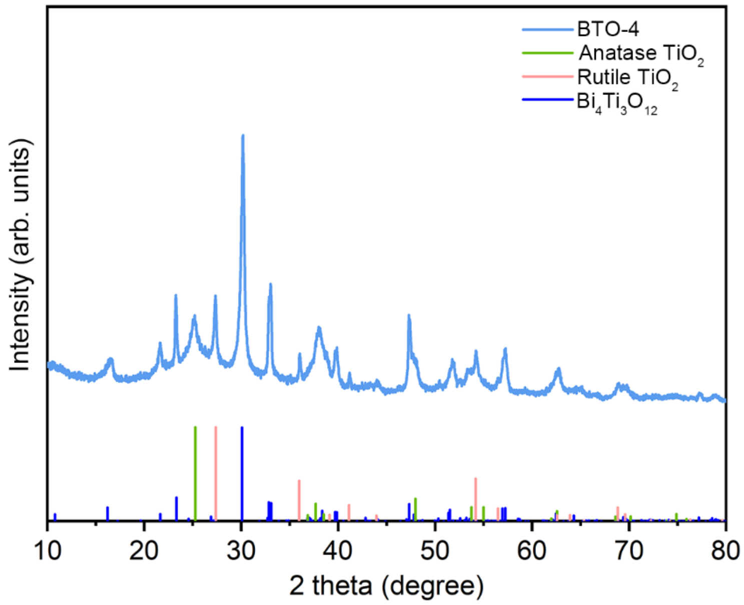

Supplementary Fig. 5. The XRD pattern of HCl treated duration for 4 h.

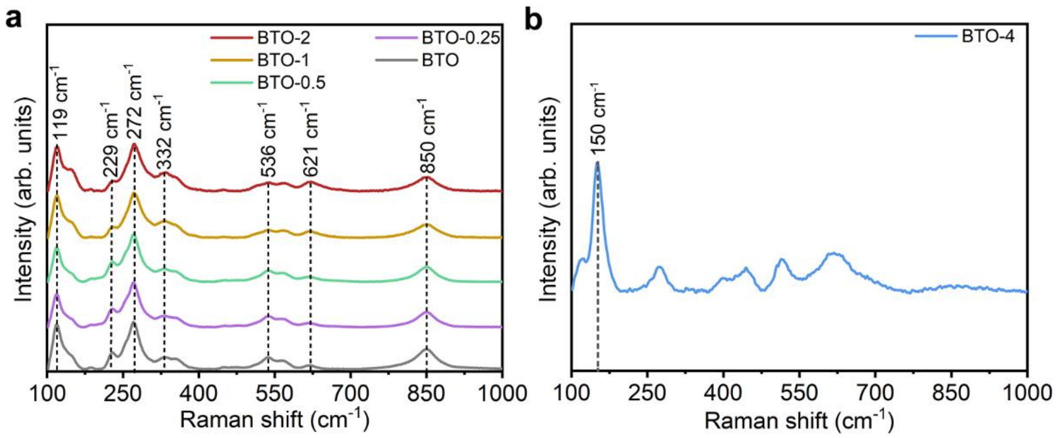

Supplementary Fig. 6. The Raman spectra of different HCl treated duration.

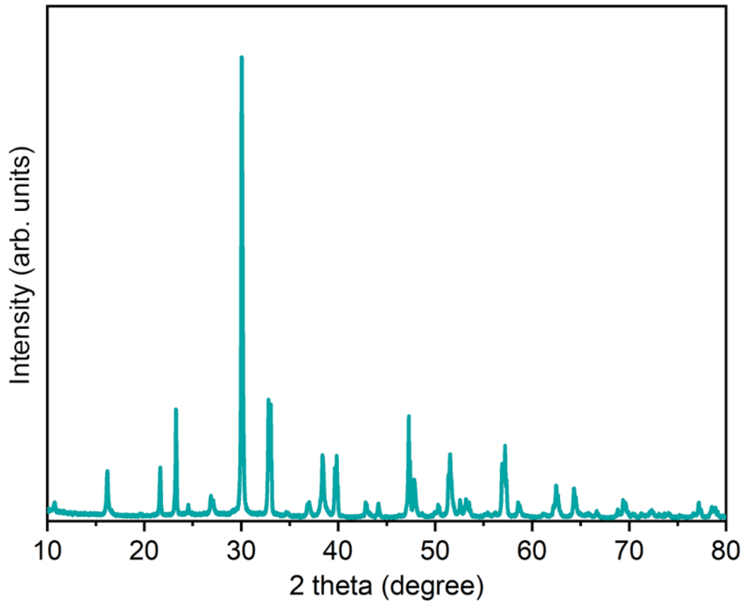

Supplementary Fig. 7. The XRD pattern of HBr treated duration for 2 h.

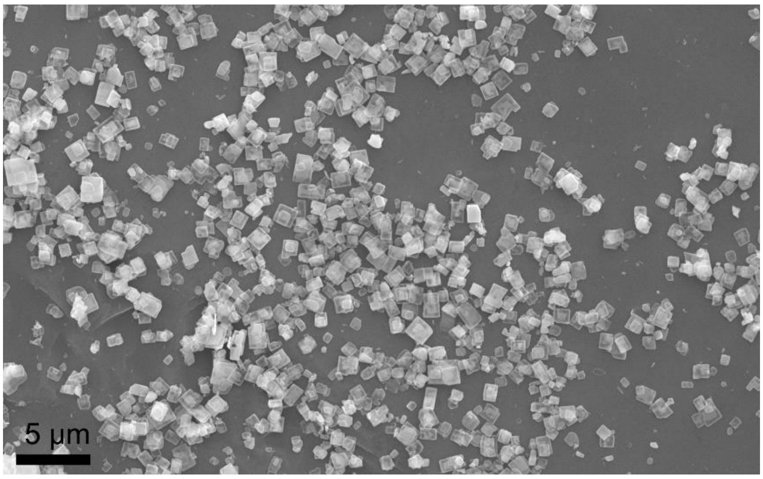

Supplementary Fig. 8. The SEM image of HBr treated BTO with same concentration as HCl for 2 h.

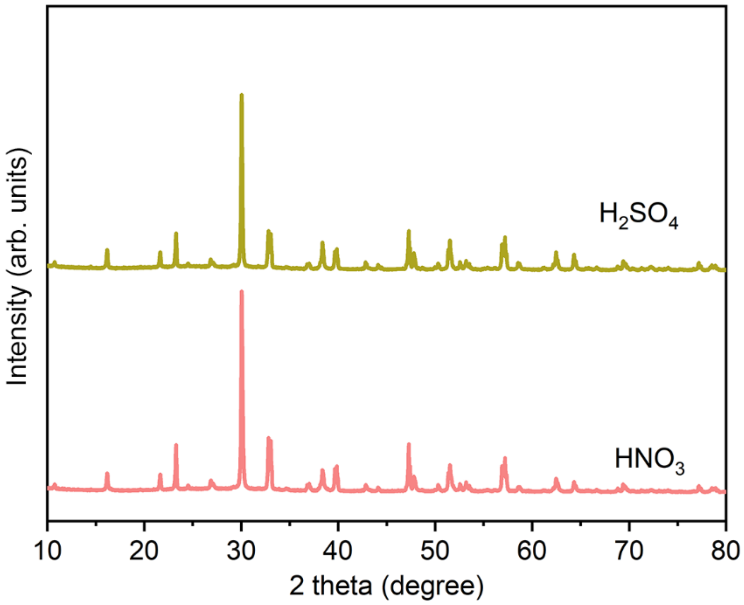

Supplementary Fig. 9. The XRD pattern of HNO_3 and H_2SO_4 treated BTO with same concentration as HCl for 2 h.

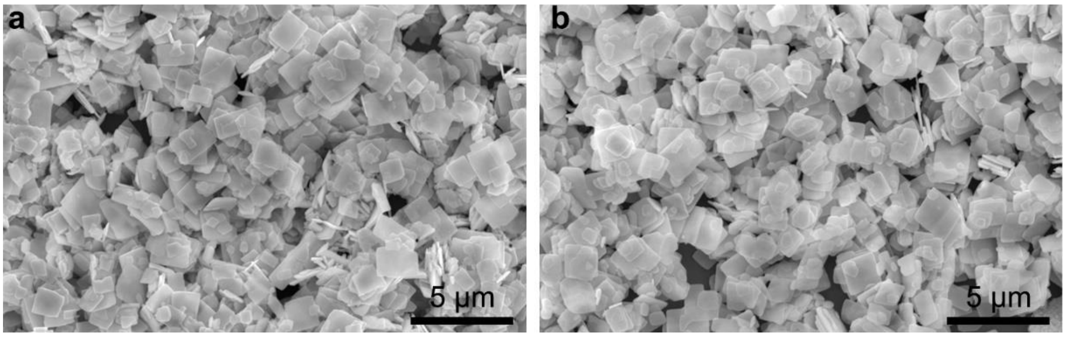

Supplementary Fig. 10. The SEM images of **a**) HNO_3 and **b**) H_2SO_4 treated with same concentration as HCl for 2 h.

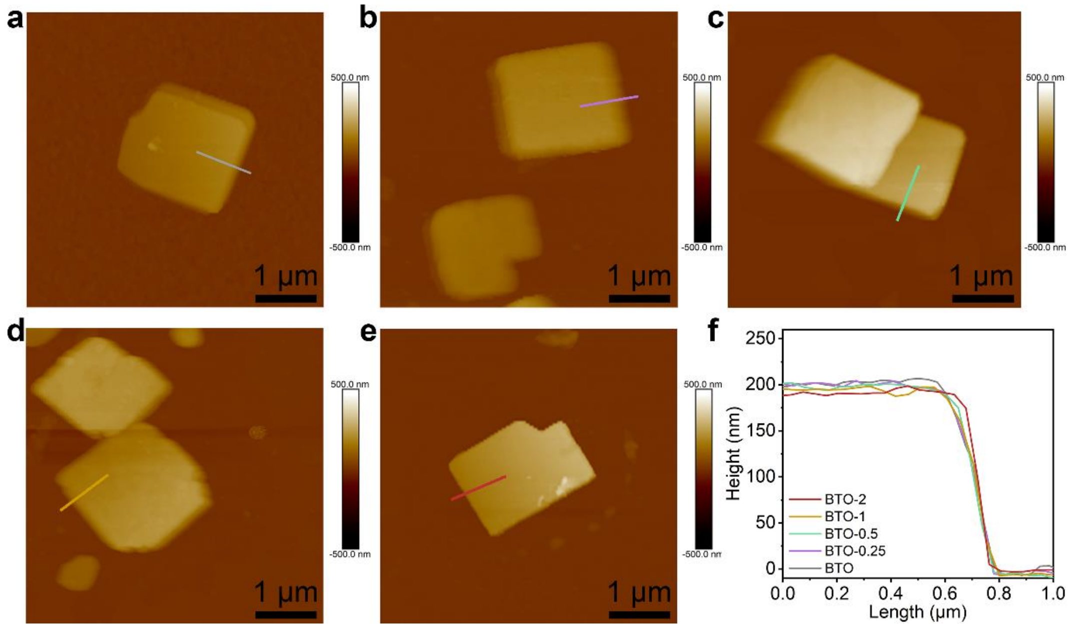

Supplementary Fig. 11. The AFM image with different etching time of BTO: **a**) BTO, **b**) 0.25 h, **c**) 0.5 h, **d**) 1 hours, and **e**) 2 h, **f**) the thickness comparison of underlined area in **a**–**e**.

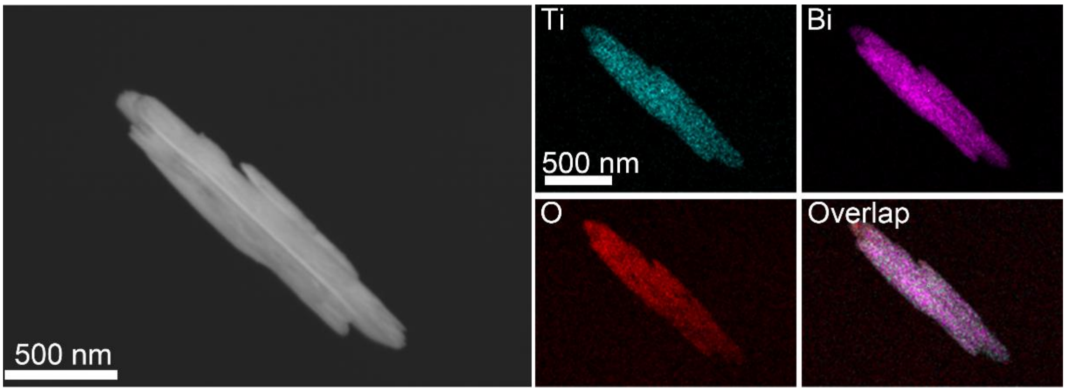

Supplementary Fig. 12. SEM image and corresponding elemental distribution of side view of BTO-2.

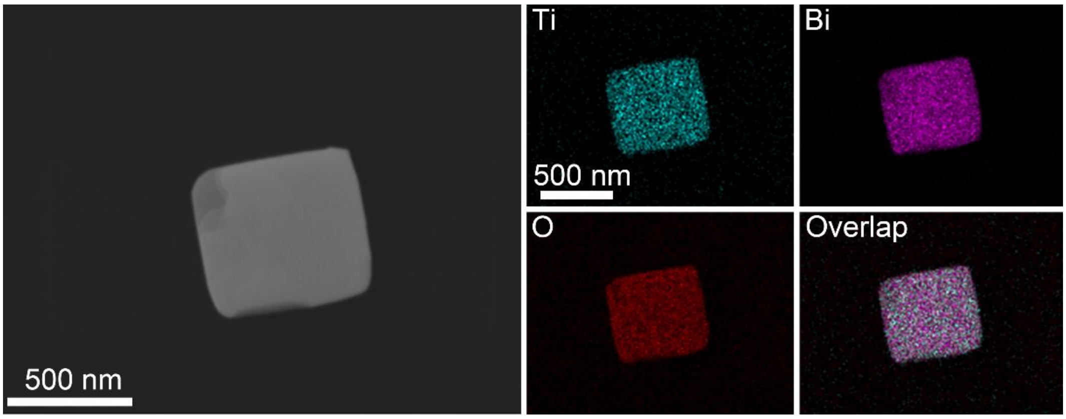

Supplementary Fig. 13. SEM image and corresponding elemental distribution of BTO.

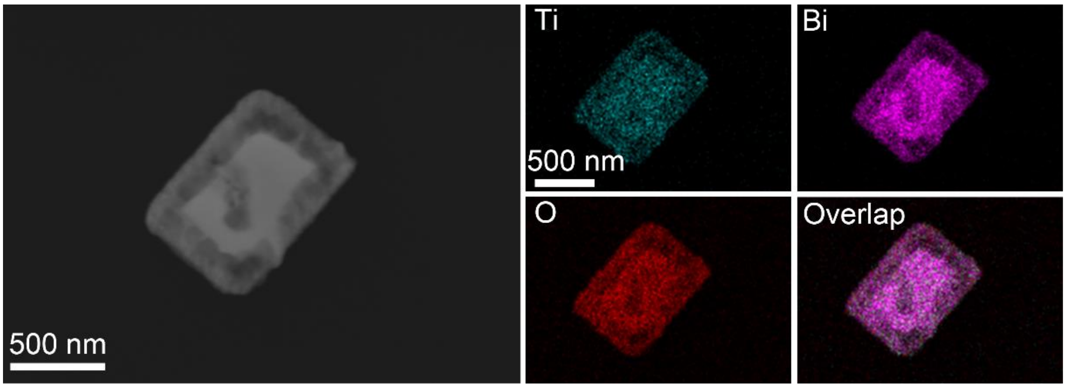

Supplementary Fig. 14. SEM image and corresponding elemental distribution of BTO-2.

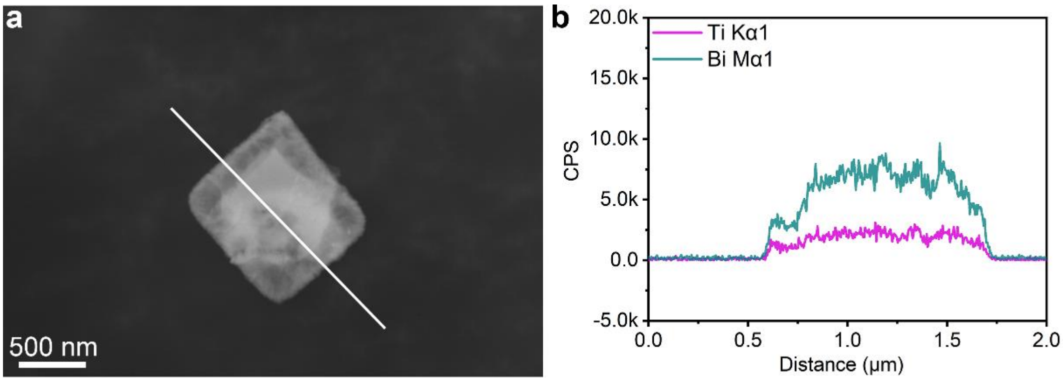

Supplementary Fig. 15. a) SEM image and **b**) corresponding EDS linear scan of BTO-2.

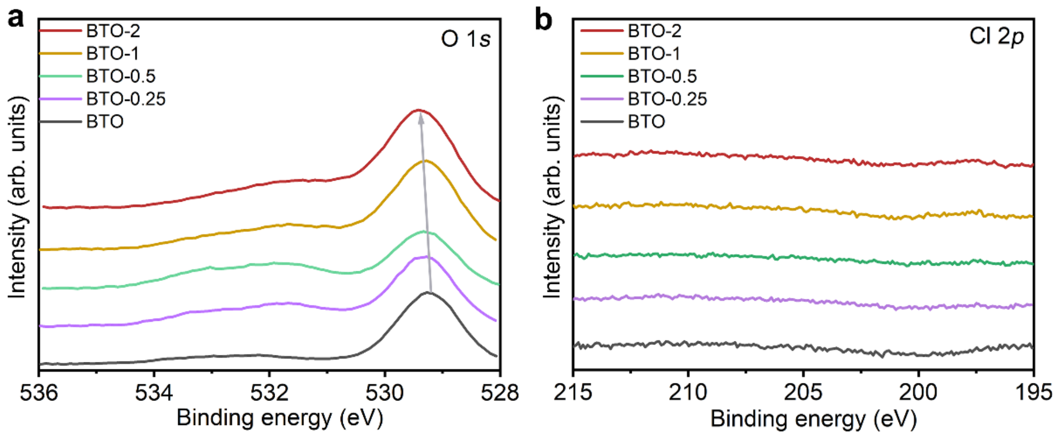

Supplementary Fig. 16. High resolution XPS spectra of **a**) O 1*s* and **b**) Cl 2*p* of BTO at different HCl treated duration.

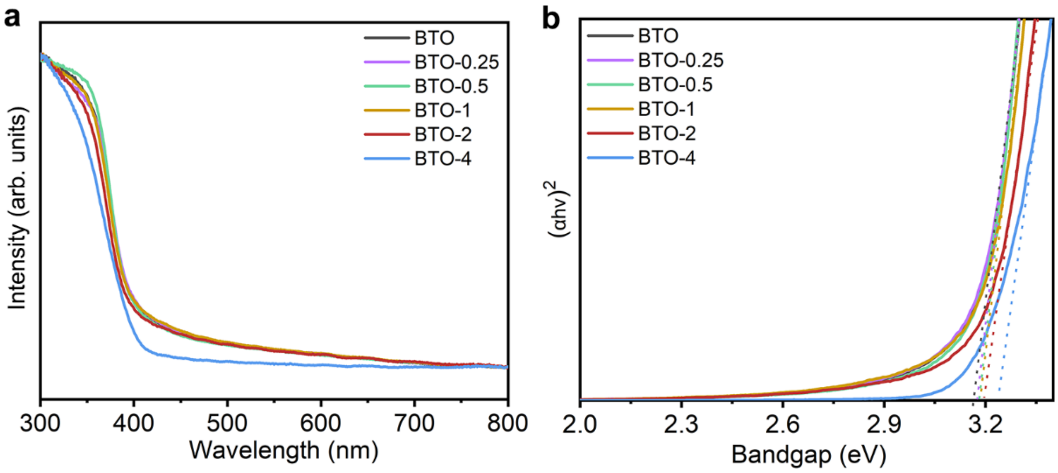

Supplementary Fig. 17. a) UV–Vis spectra and **b**) corresponding bandgap of BTO at different HCl treated duration.

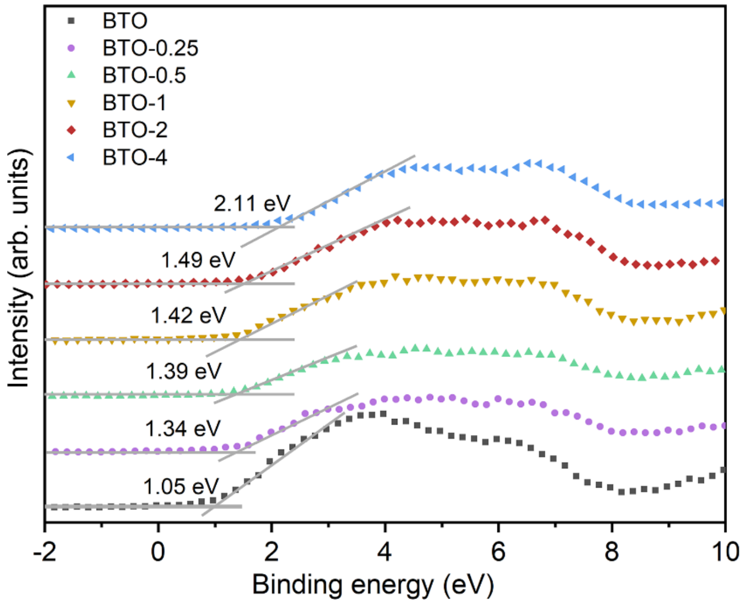

Supplementary Fig. 18. The VB-XPS spectra of BTO at different HCl treated duration.

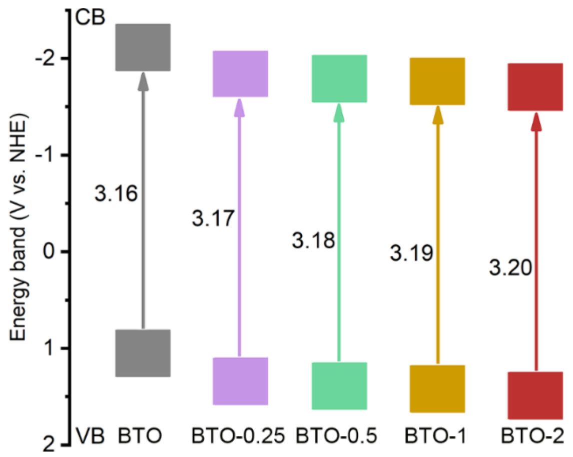

Supplementary Fig. 19. The energy band scheme of BTO at different HCl treated duration.

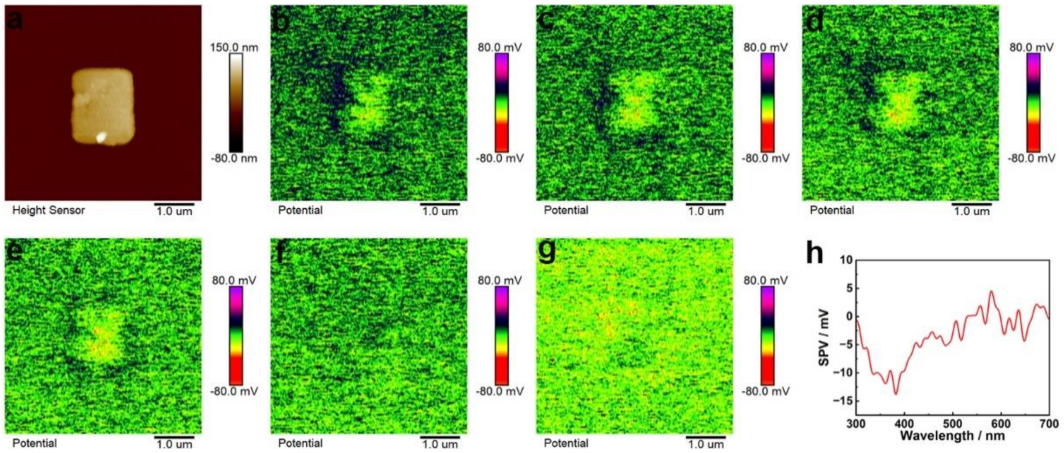

Supplementary Fig. 20. a) AFM image of BTO. **b–g**) SPVM images of BTO at different wavelengths of 350 nm, 360 nm, 370 nm, 380 nm, 400 nm, and 420 nm. **h**) SRSPS of BTO nanoparticle as a function of photoexcited wavelength under illumination.

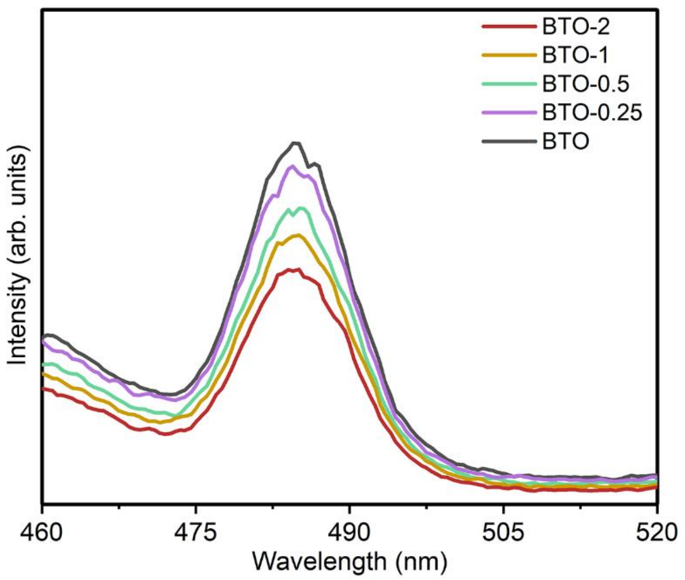

Supplementary Fig. 21. The PL spectra of BTO at different HCl treated duration.

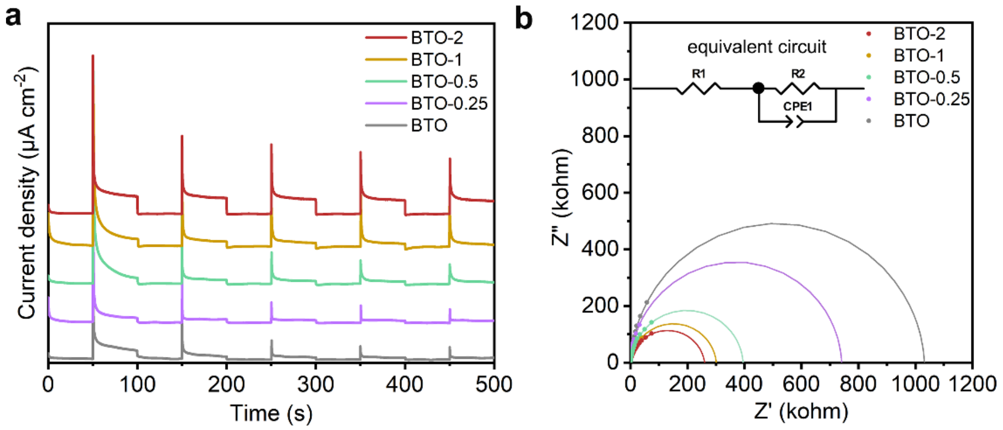

Supplementary Fig. 22. a) The photocurrent responses and **b**) EIS plots of BTO at different HCl treated duration.

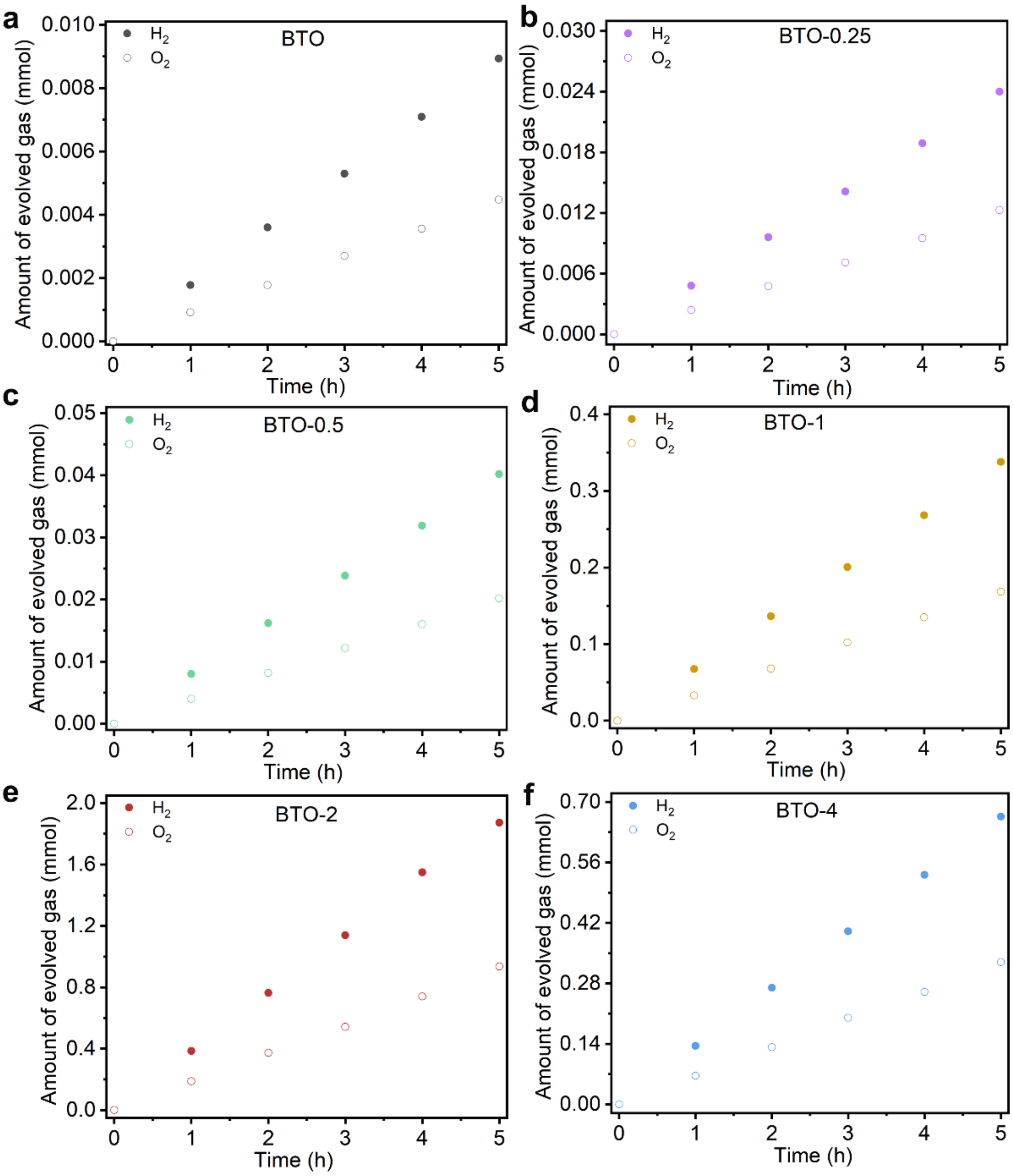

Supplementary Fig. 23. a–f) Photocatalytic overall water splitting of BTO in different etching time evolution processes.

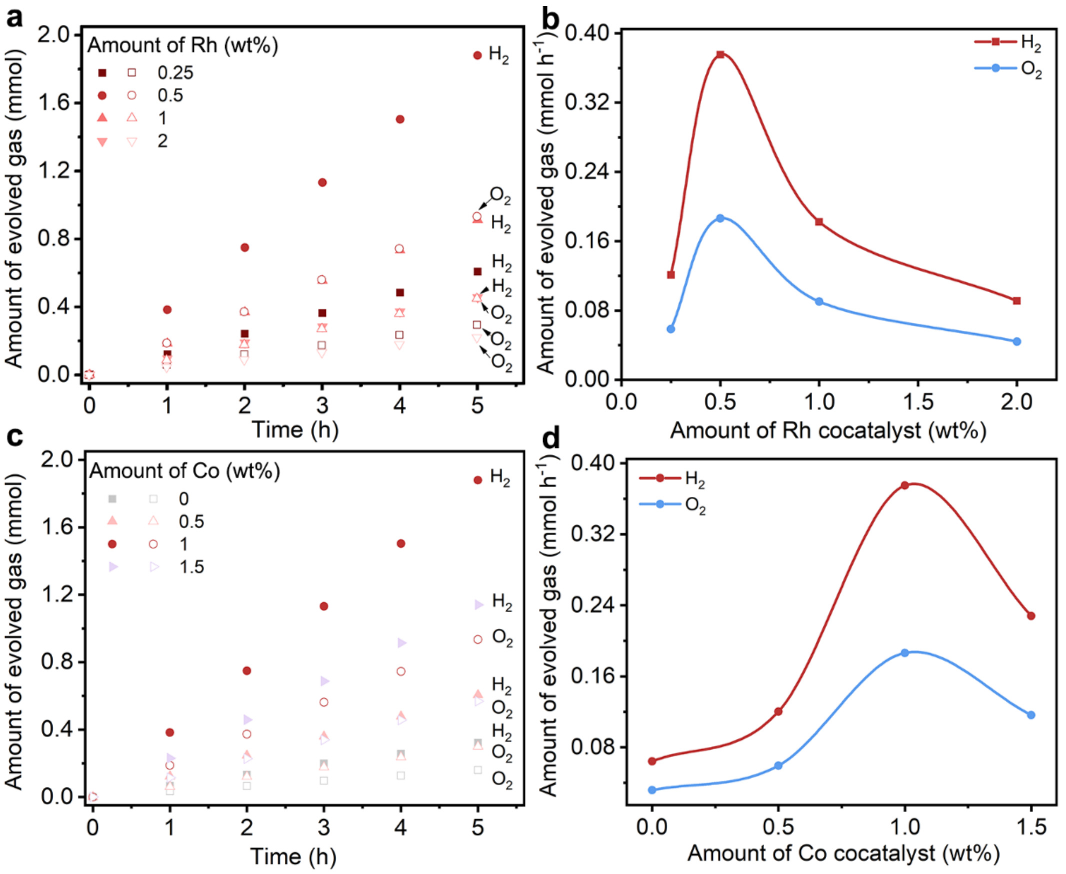

Supplementary Fig. 24. a and **b**) Photocatalytic overall water splitting of BTO-2 with different amount of photo-deposited Rh. **c** and **d**) Photocatalytic overall water splitting of BTO-2 with different amount of photo-deposited Co under UV–Vis light.

Supplementary Tables

Supplementary Table 1. The particle size, crystallite size, and BET specific surface area of BTO-2, BTO-1, BTO-0.5, BTO-0.25, and BTO.

Samples	Particle size (µm)	Crystallite size (µm)	specific surface area (m^2 g^{-1})
**BTO**	1.09	1.09	3.6
**BTO-0.25**	1.11	1.11	3.6
**BTO-0.5**	1.10	1.10	4.2
**BTO-1**	1.08	1.08	4.9
**BTO-2**	1.10	1.10	23.3

Supplementary Table 2. Comparison of the STH and produced hydrogen under xenon lamp over the BTO photocatalyst with those of reported one-excitation photocatalysts.

Materials	H_2 (μmol/h, xenon lamp)	STH (%)	Reference number
**$Bi_4Ti_3O_{12}$**	378	0.1	**This work**
**$Y_2Ti_2O_5S_2$**	7.5	0.007	Ref. 1
**Zr-TaON**	15	0.009	Ref. 2
**Organolead iodide**	3.1	0.014	Ref. 3
**Mg-$BaTaO_2N$**	2	0.0004	Ref. 4
**Ta_3N_5 nanorod**	11	0.014	Ref. 5
**$ZnIn_2S_4$**	56.6	0.003	Ref. 6
**Conjugated Polymer**	10.9	0.0047	Ref. 7
**$SrTaO_2N$**	9.1	0.0063	Ref. 8
**β-ketoamine COF**	2	0.23	Ref. 9

Supplementary Notes

Supplementary Note 1. The stability of the $Bi_4Ti_3O_{12}$ can be understood by the dissolution rate of oxide surface. For comparison between Bi–O and Ti–O termination, free energy change of metal (Bi or Ti) dissolution and oxygen dissolution on (001) and (010) surface were investigated regarding metal or oxygen on the surface. Bi^{3+} and Ti^{4+} were considered as the acid etching product. The energy of Bi^{3+} and Ti^{4+} are corrected based on standard redox potential. The theoretical stability can be described by the reaction free energy of the most thermodynamically unfavorable elementary step.

Two pathway describing catalyst dissolution with different priority

M-Path	Elementary steps
R1	Perfect → Defect_Bi_{vac} + $Bi^{\#}$
R2	Defect_Bi_{vac} + H^+ → Defect_(Bi_{vac}+O_{vac})–OH
R3	Defect_(Bi_{vac}+O_{vac})–OH + H^+ → Defect_(Bi_{vac} + O_{vac}) + H_2O

O-Path	Elementary steps
R4	Perfect + H^+ → (Defect_O_{vac})–OH
R5	Defect_O_{vac}–OH + H^+ →Defect_O_{vac} + H_2O
R6	Defect_O_{vac} → Defect_(Bi/Ti_{vac} + O_{vac}) + Ti/$Bi^{\#}$

Bi^{3+} and Ti^{4+} were considered as the acid etching product.

Supplementary Note 2. Raman is a surface test, which can detect the samples only about 10 nm. And the sample uniformity has a great impact on the results. As shown in Figure S6a, internal vibration (272, 332, 536, 621, and 850 cm^{-1}) and weak bending vibration (265 and 226 cm^{-1}) of O–Ti–O in the TiO_6 octahedra. And the lower wavenumbers at 119 cm^{-1} is originated from the vibrations between Bi and O atoms. With etching time increasing to 2 h, the Raman peaks become wider, which could be attributed to the partial etching. When the etching is 4 h, an obvious peak located at 150 cm^{-1} corresponding to TiO_2 (Supplementary Fig. 6b).

Supplementary references

1. Wang, Q. et al. Oxysulfide photocatalyst for visible-light-driven overall water splitting. *Nat. Mater.* **18**, 827-832 (2019).
2. Xiao, J. et al. Enhanced overall water splitting by a zirconium-doped TaON-based photocatalyst. *Angew. Chem. Int. Ed.* **61**, e202116573 (2022).
3. Song, X. et al. Overall photocatalytic water splitting by an organolead iodide crystalline material. *Nat. Catal.* **3**, 1027-1033 (2020).
4. Li, H. et al. One-step excitation overall water splitting over a modified Mg-doped $BaTaO_2N$ photocatalyst. *ACS Catal.* **12**, 10179-10185 (2022).
5. Wang, Z. et al. Overall water splitting by Ta_3N_5 nanorod single crystals grown on the edges of $KTaO_3$ particles. *Nat. Catal.* **1**, 756-763 (2018).
6. Pan, R. et al. Two-dimensional all-in-one sulfide monolayers driving photocatalytic overall water splitting. *Nano Lett.* **21**, 6228-6236 (2021).
7. Bai, Y. et al. Photocatalytic overall water splitting under visible light enabled by a particulate conjugated polymer loaded with palladium and iridium. *Angew. Chem. Int. Ed.* **61**, e202201299 (2022).
8. Chen, K. et al. Overall water splitting by a $SrTaO_2N$-based photocatalyst decorated with an Ir-promoted Ru-based cocatalyst. *J. Am. Chem. Soc.* **145**, 3839-3843 (2023).
9. Yang, Y. et al. Engineering β-ketoamine covalent organic frameworks for photocatalytic overall water splitting. *Nat. Commun.* **14**, 593 (2023).
